# Local approach to attributable disease burden: a case study on air pollution and mortality in Belgium

**DOI:** 10.1186/s12889-025-23625-z

**Published:** 2025-07-12

**Authors:** Arno Pauwels, Claire Demoury, Eva M. De Clercq, Brecht Devleesschauwer

**Affiliations:** 1 Department of Chemical and Physical Health Risks, Sciensano, Victor Hortaplein 40, Sint-Gillis, 1060 Belgium; 2 Department of Epidemiology and Public Health, Sciensano, Rue Ernest Blerot 1, Anderlecht, 1070 Belgium; 3 Department of Translational Physiology, Infectiology and Public Health, Ghent University, Salisburylaan 133, Merelbeke, 9820 Belgium

**Keywords:** Burden of disease, Health impact assessment, Mortality, Air pollution, Local approach, Methodology

## Abstract

**Background:**

Burden of disease estimation and the attribution to risk factors are commonly done on national or regional scale. This research proposes a novel approach, where air pollution-related mortality in Belgium was estimated locally, and compares the results to those of the common ‘global’ approach.

**Methods:**

In the local approach, mortality attributable to long-term exposure to particulate matter < 2.5 μm (PM_2.5_) and nitrogen dioxide (NO_2_) is derived at the level of census tracts. Relying on a statistical concentration-response function suggests potential bias when applied to such small scale. Therefore, the local method is validated by comparing aggregated results to estimates derived with a global approach. In a sensitivity analysis, the difference between the global and local approach is compared to the impact of other methodological choices and sources of uncertainty.

**Results:**

The local method estimates (95% confidence interval) 12,276 (6,695; 17,826) deaths for PM_2.5_ and 7,944 (4,725; 11,181) for NO_2_ in Belgium. For both pollutants, these national estimates never deviate more than 2% from those obtained with the global method, and never more than 4% in the individual provinces. The sensitivity analysis demonstrates the concentration-response function as having the largest contribution to overall uncertainty, while the global-local discrepancy is slightly larger compared to the exposure uncertainty.

**Conclusions:**

Aggregated local burden estimates prove to be accurate compared to the global approach. This means the local method shows potential for comparing areas and population groups at subnational level, where estimates can be generated in a flexible manner depending on research or policy needs.

**Supplementary Information:**

The online version contains supplementary material available at 10.1186/s12889-025-23625-z.

## Background

Burden of disease studies aim to create a comprehensive and coherent framework to quantify the impact of physical and psychological diseases, injuries and risk factors. Such estimates are crucial in health impact assessment, as they allow to put numbers on the effect of proposed public health policy scenarios [1]. Disease burden estimates can be attributed to a risk factor by means of comparative risk assessment (CRA), as to gain insight in the relative importance of different health determinants and their impact on population health. CRA is a top-down method, relying on an assessment of population exposure and the relative risk (RR) of an associated health outcome to estimate the share of the disease burden attributable to the risk factor (also known as the population attributable fraction, or PAF). This method is suited to assess the burden attributable to environmental determinants of health, such as air quality [2].

Despite being widely applied, the CRA methodology as such has not received great attention in literature. Attention has gone to different approaches for calculating population attributable fractions (e.g [3, 4]).,, adjustment and stratification (e.g [5]).,, determining uncertainty and confidence intervals (e.g [6, 7]).,, interpretation of PAFs (e.g [8, 9]).,. One often ignored challenge is that of heterogeneity, as might exist across geographic areas (e.g., districts, municipalities or census tracts) or population subgroups (e.g., based on age, sex or socio-economic status). In the usual approach for attributable disease burden calculation, a PAF is determined for the area of interest (usually a country or a region) and multiplied with the total burden for that area. In the case of the burden attributable to air pollution, the PAF is derived based on summary estimate of population exposure, usually a population-weighted average concentration.

By relying on such a ‘global’ approach, the methodology neglects any variation that might exist in exposure and underlying burden. A possible way to navigate this heterogeneity is to pursue a local approach to estimate attributable disease burden. The core idea is that the complete chain of calculations, from exposure assessment, over PAFs to attributable burden, is conducted on small area-level, such as census tracts. The resulting local burden results can then be aggregated to obtain estimates for wider regions and populations. This bottom-up approach allows to map the attributable mortality and inspect local differences between areas and population subgroups.

The objective of this paper is to compare two different approaches to estimating the national burden of disease attributable to air pollution. The selected risk factors are long-term exposure to ambient particulate matter with an aerodynamic diameter ≤ 2.5 μm (PM_2.5_) and nitrogen dioxide (NO_2_), and the studied outcome is all-cause mortality. The case was developed for Belgium, a densely populated country in Western Europe experiencing high levels of air pollution [10, 11]. Poor air quality in Belgium is responsible for increases in mortality [12], general practitioner and emergency room visits [13] and depressive disorder diagnoses [14]. Although a global approach is more rigid compared to the local approach, the former can be considered more robust in the sense that the estimate is based on the burden in large population groups. To test for any bias, the local estimates are aggregated to different subnational levels and compared to estimates generated with a global approach. In a sensitivity analysis, the discrepancy between the local and global approach is compared to the variance of the estimates resulting from other methodological choices and the impact of different sources of uncertainty.

## Methods

### Local and global approach

The local and global approach to attribute mortality to air pollution are both implementations of CRA. In summary, CRA combines an estimate of population exposure to a given risk factor with the relative risk of an associated health outcome, to obtain a population attributable fraction, or PAF. The PAF corresponds to the proportion of the total disease burden that is attributable to the risk factor in question. The PAF as a fraction is multiplied with the observed burden of disease to yield an estimate in absolute terms. Population exposure assessment can be based on measured or modelled air quality, while the risk is calculated using a concentration-response function (CRF) taken from the epidemiologic literature.

Burden of disease studies are usually conducted at a wide scale, typically on a national or regional level. In the case of air pollution, exposure in the population is often summarised by means of a population-weighted average concentration. This value represents the mean concentration in a territory (e.g., a country), where the calculation takes the population number at each location (e.g., a census tract) as a weight. Population weighting aims to avoid a biased exposure estimate, which would occur if the population is not homogeneously distributed across the territory, i.e., if the population resides in areas that are on average either more or less polluted compared to the spatial average concentration in the territory. The methodological choice for either a population-weighted or (unweighted) spatial average concentration to assess population exposure will impact the final burden estimate.

The risk of all-cause mortality is calculated with a CRF. A CRF yields the relative risk of the outcome in function of exposure to the risk factor, in this case the ambient concentration of PM_2.5_ and NO_2_. This function is usually sourced from a single epidemiolocal study, or from a meta-analysis that combines effect measures from multiple publications that study the same association. The population or populations included in the study or meta-analysis are assumed to be representative for the ‘target’ population, in this study the inhabitants of Belgium in 2019. As such, a CRF allows to derive the RR of all-cause mortality in Belgium based on the existing level of population exposure. This RR value is then contrasted with a counterfactual situation where population exposure is minimal, corresponding to no increased risk (i.e., a RR equal to 1). Doing so allows to obtain the fraction of all deaths attributable to PM_2.5_ and NO_2_, i.e., the PAF.

In contrast to the country- or region-wide scale, ‘global’ approach, we propose a local approach to estimate the burden of disease attributable to risk factors. In the local approach, the steps of CRA are carried out on a small-area level, instead of national or regional level. Local population exposure and corresponding relative risk are determined, and the PAF determines the attributable burden on the same scale. The local results can then be aggregated to obtain estimates at wider areas and larger populations. As a case study, mortality from all causes attributable to PM_2.5_ and NO_2_ is determined locally, on the level of census tracts (also referred to as statistical sectors), in Belgium in the year 2019. The local results are aggregated and compared with estimates obtained using a global approach, to verify any potential systematic bias of local estimates compared to the global approach. A sensitivity analysis is conducted to compare the discrepancy in estimates of the two approaches to any discrepancies resulting from methodological choices and to the uncertainty related to different inputs. The analysis was carried out in R version 4.2.3.

### Baseline mortality data

The health outcome in this study is mortality from all causes (both natural and external causes). The deaths in 2019 are extracted from the national mortality register, maintained by Statbel, the Belgian statistical office. The data concern individual-level records, containing the deceased person’s statistical sector of residence. Statistical sectors are census tracts defined by Statbel, a subdivision of the municipality based on socio-economic and urban morphological characteristics, with the aim of reporting statistics at a more refined scale. The statistical sectors were established in 1970 and were revised several times since to account for changes in population and land use [15].

**Fig. 1:**
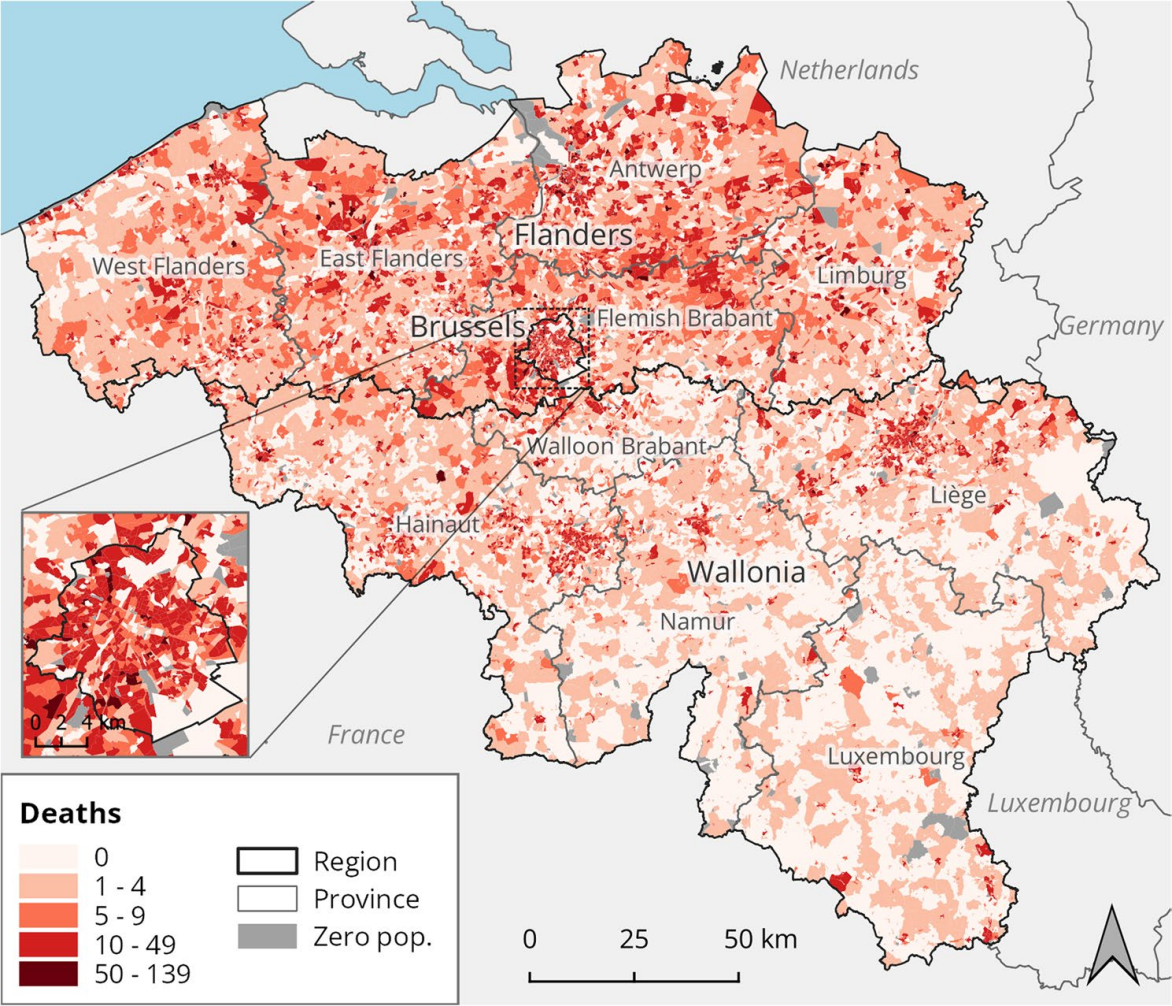
Deaths by statistical sector in Belgium, 2019. Sectors with no population, indicated in grey, are excluded from the analysis. Data from the Belgian mortality register, provided by the Belgian Statistical Office

Given the availability of mortality data at this scale, the statistical sector was used as the geographical unit of analysis in the local approach to estimate air pollution attributable burden. In the 2020 definition, there are a total of 19,794 statistical sectors, of which 19,414 (98%) are inhabited. The population in these sectors ranges from 1 to 8,340 inhabitants, with a median of 336, in 2019. Mortality for the year 2019 ranges from no deaths (in 4746 or 24% of sectors) to 139 deaths, with a median of 2 deaths per sector (Table S1). The number of deaths in each statistical sector serves as baseline burden in the local approach (Fig. 1). In the subsequent analyses, only the inhabited sectors are considered.

A global approach requires mortality counts for Belgium as a whole or for subnational regions. For this end, the statistical sectors are aggregated to larger areas, where the area’s mortality count is the sum of the mortality in the sectors that make up the area. For the purposes of this study, all-cause mortality was derived for the regions and provinces of Belgium, as well as a national total (Table 1). In the European NUTS classification, the regions are the first subdivision of Belgium (NUTS1). Each region can potentially be divided into provinces, marking the second NUTS subdivision (NUTS2). The Brussels-Capital Region serves as a statistical region in both the first and the second subdivision, which means it can be put on par with both the regions and the provinces.

**Table 1:** Population, deaths and mortality rate in the provinces (NUTS2), regions (NUTS1) and in total in Belgium, 2019

	Area	Population	Deaths	Mortality rate
**Province (NUTS2)**	Antwerp	1,857,435	17,018	916.21
	Walloon Brabant	403,549	3,487	864.08
	Hainaut	1,343,858	14,750	1097.59
	Liège	1,106,725	11,410	1030.97
	Limburg	873,812	7,776	889.89
	Luxembourg	284,556	2,661	935.14
	Namur	494,256	5,098	1031.45
	East Flanders	1,514,345	14,606	964.51
	Flemish Brabant	1,145,803	10,347	903.03
	West Flanders	1,195,349	12,668	1059.77
**Region (NUTS1)**	Brussels	1,205,525	8,909	739.01
	Flanders	6,586,744	62,415	947.59
	Wallonia	3,632,944	37,406	1029.63
**Country**	Belgium	11,425,213	108,730	951.67

### Exposure assessment

The risk factors selected in this study are long-term exposure to respectively PM_2.5_ and NO_2_, quantified as the annual average ambient concentration. Population exposure assessment in this study is based on high resolution air pollution raster maps provided by the Belgian Interregional Environment Agency [16]. The maps are the result of ATMO-Street, a state-of-the-art model that combines observations with data on land cover, emissions, weather conditions and building geometry, and is calibrated against actual air quality measurements.

**Fig. 2:**
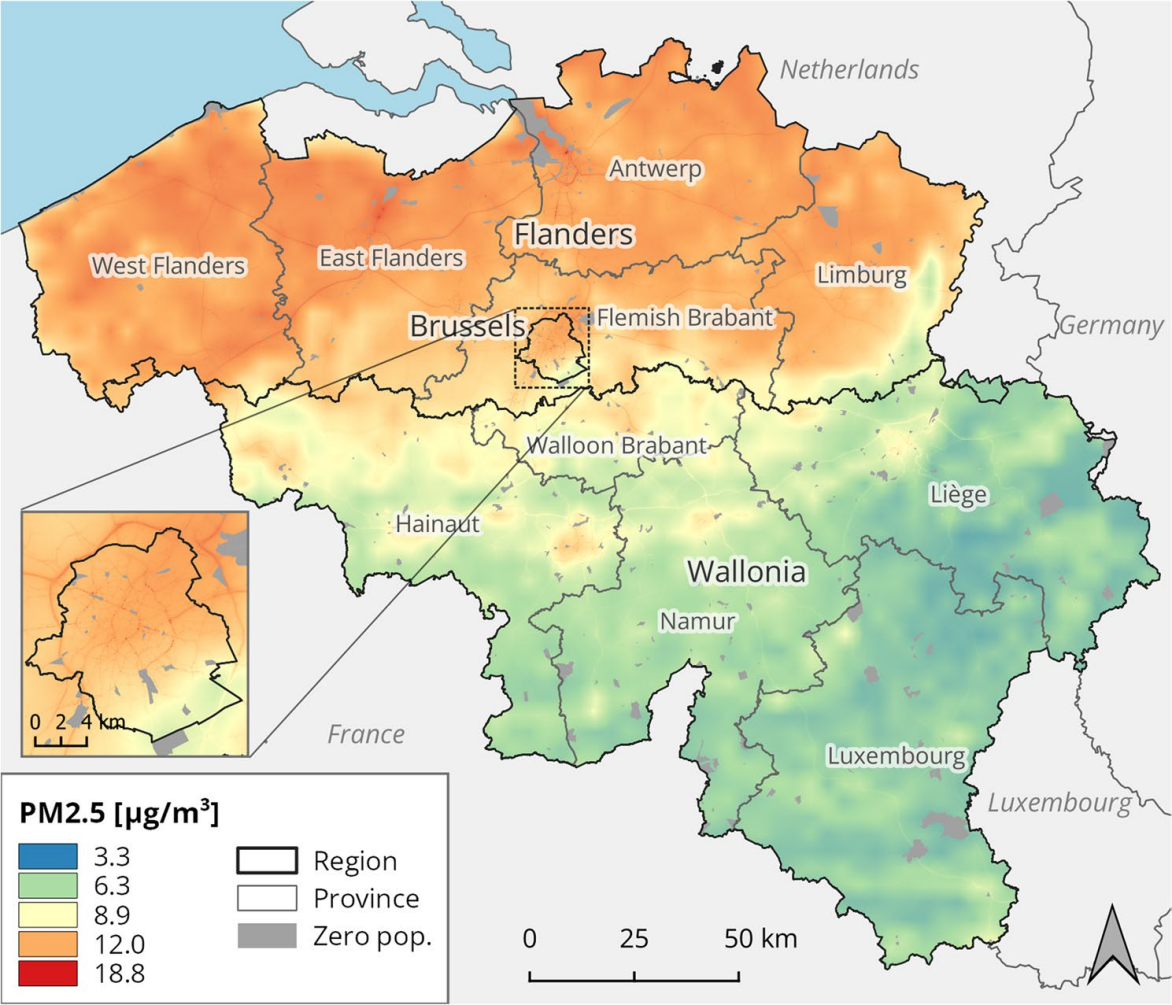
Annual average concentration of PM_2.5_ in Belgium, 2019. Statistical sectors with no population, indicated in grey, are excluded from the analysis. Results from the ATMO-Street model chain, provided by the Belgian Interregional Environment Agency

**Fig. 3:**
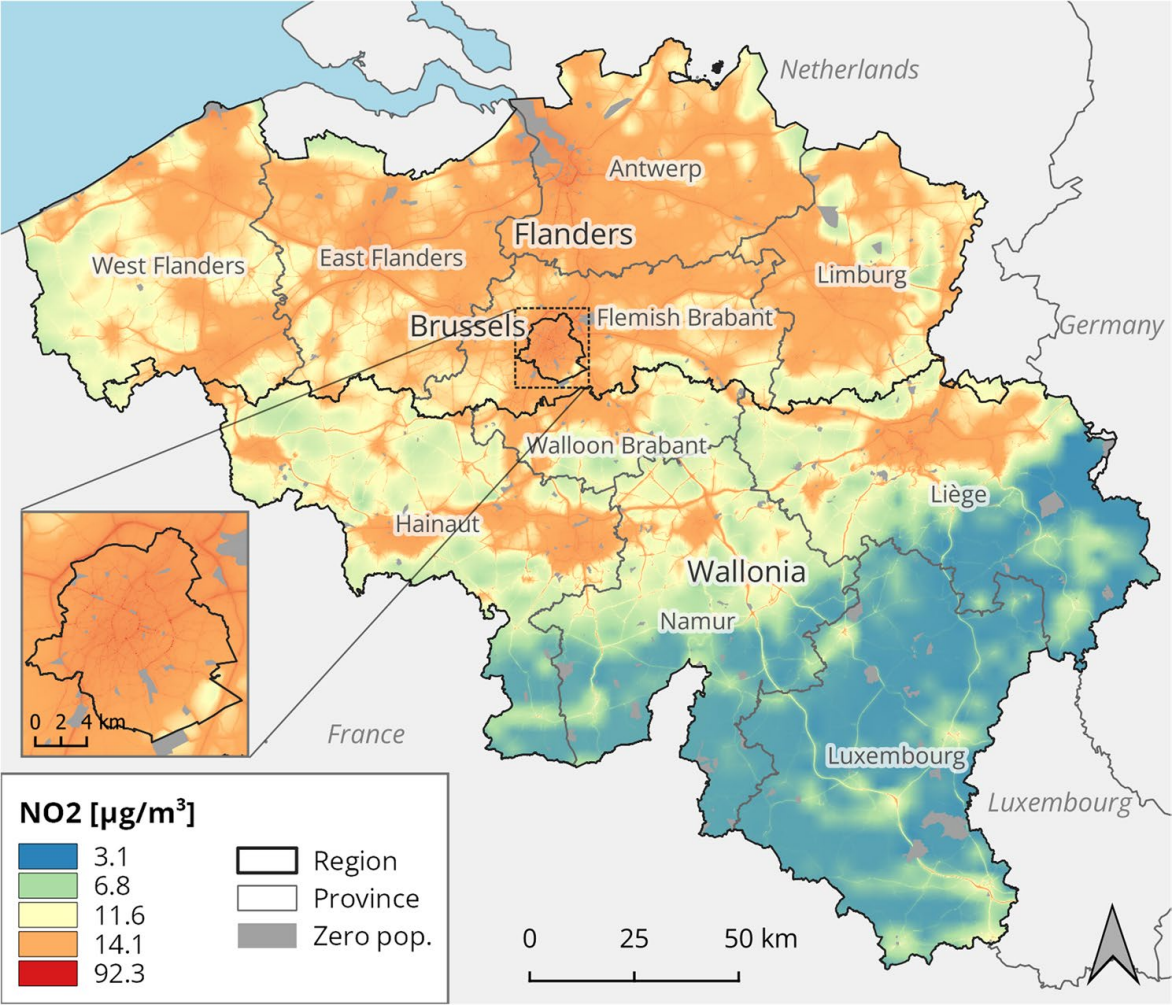
Annual average concentration of NO_2_ in Belgium, 2019. Statistical sectors with no population, indicated in grey, are excluded from the analysis. Results from the ATMO-Street model chain, provided by the Belgian Interregional Environment Agency

ATMO-Street is a chain of three air quality models [17]. Firstly, background concentrations are derived by the interpolation of observations at fixed monitoring stations, relying on the 4 × 4 km^2^ RIO (residual interpolation optimised for ozone) model. RIO accomplishes spatial interpolation in a ‘smart’ way, relying on the association between concentrations and land cover, taken from the European CORINE dataset. Secondly, pollutant dispersion is modelled with the immission frequency distribution model (IFDM). IFDM uses data on the emissions from road traffic, shipping and large industrial point sources, as well as meteorological conditions. Lastly, the effect of concentration build-up in street canyons is simulated with the operational street pollution model (OSPM), relevant for urban settings especially. Double-counting corrections are applied on the combined model results. The final ATMO-Street model is resolved on a highly refined grid with 10 m resolution (Figs. 2 and 3).

The dispersion of PM_2.5_ is diffuse, showing a regional pattern where the northern Flemish Region is generally more polluted compared to the southern Walloon Region, especially compared to the south-eastern forested and hilly part. NO_2_ concentrations are more spatially variable, exhibiting a large contrast between highly polluted urbanised areas, including the Brussels-Capital Region, and highways compared to less densely populated rural and suburban areas.

To determine population exposure to PM_2.5_ and NO_2_, the pollution raster maps were overlaid with a geographic vector map of the statistical sectors. In the global approach, the average concentration value is calculated for each sector, and these local values are then aggregated to larger areas with the population number in each sector (as a fraction of the total population in the area) serving as a weight. The result is a population-weighted average concentration, used as the exposure value for the aggregated region. In the local approach, exposure is determined as the spatial mean concentration in the statistical sector. As such, the spatial average concentration is assumed to be representative for the inhabitant’s exposure, justified by their small size and internal homogeneity.

To model the uncertainty related to population exposure, a set of mean concentrations is generated by bootstrapping. In each statistical sector, an average concentration is calculated 1,000 times by means of resampling with replacement among the raster cells within the sector. The resulting distribution is centred around the sector’s mean concentration, and the spread is determined by the variability in concentration. As such, resampling concentrations allows to model the uncertainty related to the distribution of the local population within the sector. In the local approach, this distribution represents the uncertainty in the local exposure. In the global approach, the first resampled mean concentration in all sectors is aggregated, then the second resampled mean etc., resulting in 1,000 population-weighted average concentrations.

### Concentration-response functions

The CRFs selected for this study result from the research project *Effects of Low-Level Air Pollution: A Study in Europe* (ELAPSE) [18]. At the time of analysis, this is the most recent comprehensive evidence of air pollution health impacts, analysing data from multiple conventional as well as large administrative cohorts. It includes data on about 30 million participants in 11 EU countries, including an administrative cohort from Belgium [19]. The research focuses on the European context, where pollution levels are generally lower compared to lower income countries, which renders the results relevant for the Belgian context. In a commentary piece by some of the researchers of ELAPSE, the effect estimates of the conventional and administrative cohorts are combined using a random effects model. After pooling, the authors found the following RRs (95% confidence interval) per 10 µg m^−3^ increase in exposure concentration: 1.118 (1.060; 1.179) for PM_2.5_ and 1.045 (1.026; 1.065) for NO_2_ [18]. Assuming a log-linear relationship, the *RR* in function of exposure concentration *C* is calculated as (Eq. 1):

1 $$RR\left(C\right)\;=\;exp\;\left(\frac{\ln\left(RR_{10}\right)}{10}{\times C}\right)$$

with *RR*_*1*0_ the increase in RR per 10 µg m^−3^ increase in exposure concentration, its value depending on whether PM_2.5_ or NO_2_ is considered. No exposure threshold was instated, assuming no safe level of air pollution exists. This is in line with the findings of ELAPSE, where in both the conventional as the administrative cohorts the shape of the CRF was found to be supralinear with no indication of a level below which no association was found [19].

The impact of uncertainty on the CRF was taken into account by means of parametric bootstrapping. We performed Monte Carlo simulations by random sampling from a gamma distribution fitted to the mean and 95% confidence interval of the RR_10_ for PM_2.5_ and NO_2_. For each pollutant, this resulted in a set of 1,000 RR_10_ values, which were paired with the 1,000 resampled mean concentrations obtained in the exposure assessment. The same set of RR_10_ values was used consistently for all sectors in the local approach and all aggregated areas in the global approach.

### Population attributable fraction

In case exposure is uniform in the population (either approached as a local spatial average or a global population-weighted average concentration) and the counterfactual (in this case zero pollution concentration) corresponds to a RR equal to 1, the *PAF* can be calculated as (Eq. 2):

2 $$PAF\;=\;\frac{RR\left(C\right)-1}{RR\left(C\right)}$$

with *RR(C)* the relative risk at exposure level *C*. The PAF is applied to the whole population residing in the area, regardless of age at the time of death.

Based on the 1,000 bootstrapped values of mean exposure and RR(C), 1,000 values for the PAF are calculated. As the number of deaths is extracted from the mortality register, these figures are treated as fully accurate, and the uncertainty on the final attributable mortality estimate is entirely determined by the distribution of the PAF. In the global approach, the RR is a function of the population-weighted average concentration of the region of interest. The air pollution attributable mortality can be calculated by multiplying the global PAF with the total mortality in the region. In the local method, the RR varies with the local concentration level of each statistical sector. The local air pollution attributable mortality is then calculated by multiplying the local PAF with the mortality count in each sector. These local values sum up to the attributable burden for the whole region, which yields an estimate that can be compared to the result of the global method.

### Aggregation of local estimates

The local approach to attributable disease burden yields a large number of small-scale estimates, which allows to examine variation of the burden in space and within the population. In this case, air pollution attributable mortality in Belgium was estimated at the level of the statistical sector. Aside from considering local estimates as such, the sector results can be aggregated by delineating a collection of sectors (making up e.g., a region, province, city) and summing the local burden estimates into a larger area and population total. To generate the confidence interval on the aggregated estimate, the varying estimates obtained in each iteration of the bootstrapping are summed up separately, resulting in a set of aggregated burden results. Based on this distribution, a mean and confidence interval are calculated.

In this manner, burden estimates can be obtained for any area or population that is an aggregate of statistical sectors. For the purpose of this study, aggregated air pollution attributable mortality estimates are calculated for the regions and provinces, the NUTS1 and NUTS2 subdivisions of Belgium respectively. The results of the aggregation can then be juxtaposed to air pollution attributable mortality estimates obtained with the global approach. Assuming the global estimates are accurate, the aggregated local results can be verified in this manner. If the local estimates are biased, the aggregated values would be systematically higher or lower than the corresponding estimates derived with the global approach. If there is no systematic deviation, this does not indicate that the local results are fully accurate, but at least that when aggregated to a sufficiently large population, any individual errors are overcome.

### Sensitivity analysis

Of main concern in this research is the effect of the local versus global approach on the estimated attributable burden. To compare the discrepancy between both methods to the variation resulting from other methodological choices and sources of uncertainty, a sensitivity analysis is performed. In the analysis, the mortality attributable to PM_2.5_ and NO_2_ is estimated for Belgium applying the global approach, and compared to the estimate obtained by aggregating the local results. The discrepancy between the local and global estimates is contrasted to the total and source-specific uncertainty on the estimates, and to the effect of methodological assumptions.

The global (central) estimate serves as the baseline in the sensitivity analysis. The total uncertainty on the global estimate, resulting from exposure and CRF uncertainty, is calculated using bootstrapping as previously described. Aside from this baseline, four alternative scenarios were investigated (Table 2). In a first scenario, the local approach was used to estimate air pollution attributable burden. Attributable mortality was computed for each statistical sector, and subsequently summed to a Belgian total. The difference between the baseline and the local estimate reflects the sensitivity to the choice for a local approach. In a second scenario, a global approach with an unweighted spatial average concentration was used as the population exposure value. Any deviation from the baseline marks the impact of the methodological choice for population-weighting in the exposure assessment. To gain insight in the contribution of population exposure and the CRF to the total uncertainty on the air pollution mortality estimates, two last scenarios were constructed. In one, the resampled exposure values were combined with a constant RR equal to its mean value, resulting in a confidence interval reflecting the contribution of population exposure uncertainty. The uncertainty on the concentration values (as a result of model error) is as such not accounted for. In the other scenario, population exposure was kept equal to the mean, resulting in a confidence interval reflecting the simulated uncertainty on the selected CRF. The effect of applying other potential CRFs is not tested in the sensitivity analysis. In case of any deviation between the baseline and the central estimate in the scenarios with constant exposure or constant risk, the confidence intervals were shifted in a way the central estimate and baseline coincide.

**Table 2:** Definition of the baseline and alternative scenarios used in the sensitivity analysis

Scenario	Definition
Global approach (baseline)	Global approach with population-weighted average concentration. Central estimate with lower and upper bound of total uncertainty (CRF and exposure assessment).
Local approach	Local approach. Central estimate of aggregated results.
Spatial average concentration	Global approach with spatial average concentration. Central estimate.
Exposure uncertainty	Global approach with population-weighted average concentration. Varying population exposure (resampling) with central RR estimate.
Risk uncertainty	Global approach with population-weighted average concentration. Varying RR (Monte Carlo simulation) with central population exposure estimate.

## Results

**Fig. 4:**
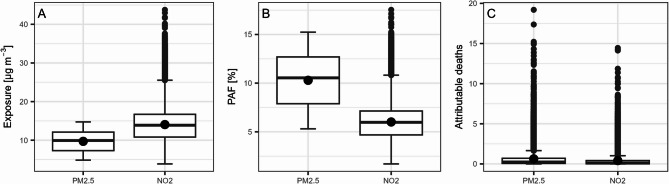
Boxplots summarizing the local estimates of PM_2.5_ and NO_2_ (**A**) exposure, (**B**) population attributable fraction and (**C**) attributable mortality in Belgium (*N* = 19,414). Mean and median, outlier > 1.5 IQR from median

The boxplots in Fig. 4 summarize local air pollution exposure, attributable fractions and mortality estimates (Tables S2 & S3). Exposure to both PM_2.5_ and NO_2_ varies greatly within the country, where local concentrations can deviate substantially from the mean (Fig. 4 A). While exposure to PM_2.5_ ranges from approximately 5 to 15 µg m^−3^, NO_2_ exposure starts at about 3 µg m^−3^ and can reach values to above 40 µg m^−3^ on some locations. This illustrates the relatively high spatial variation of the dispersion of NO_2_. These exposure ranges are covered by the ELAPSE study, making it appropriate to use the CRFs for the Belgian population. In the pooled conventional cohort, exposure ranges from 3.24 to 27.49 µg m^−3^ for PM_2.5_, and from 2.68 to 81.02 µg m^−3^ for NO_2_; in the administrative cohorts, exposure ranges from 0 to 27.96 µg m^−3^ for PM_2.5_, and from 0.17 to 93.76 µg m^−3^ for NO_2_.

As the PAF is a function of exposure, the variation in local concentrations is reflected in the range of population attributable fractions (Fig. 4B). The statistical sector’s mean PAF and standard deviation respectively are 10.3% and 2.6% for PM_2.5_ vs. 6.0% and 2.3% for NO_2_ (Table S1). The PAFs are generally higher for PM_2.5_ compared to NO_2_, an expected result given the steeper RR gradient for the former. The high outlying exposure concentrations for NO_2_ result in a number of extreme PAFs. As a result, the highest PAFs are observed for NO_2_.

As total mortality is generally low in individual statistical sectors, air pollution-attributable mortality is limited as well (Fig. 4 C). This is related to the small size and populations of the sectors, with a median of 2 deaths, and zero deaths in almost a quarter of the sectors (Table S1). The third quartile is below one case for both pollutants, meaning that in the majority of sectors there is less than one attributable death per year. This result highlights the fact that the local attributable estimates need to be interpreted with care. Mortality attributable to PM_2.5_ is higher compared to NO_2_.

**Fig. 5:**
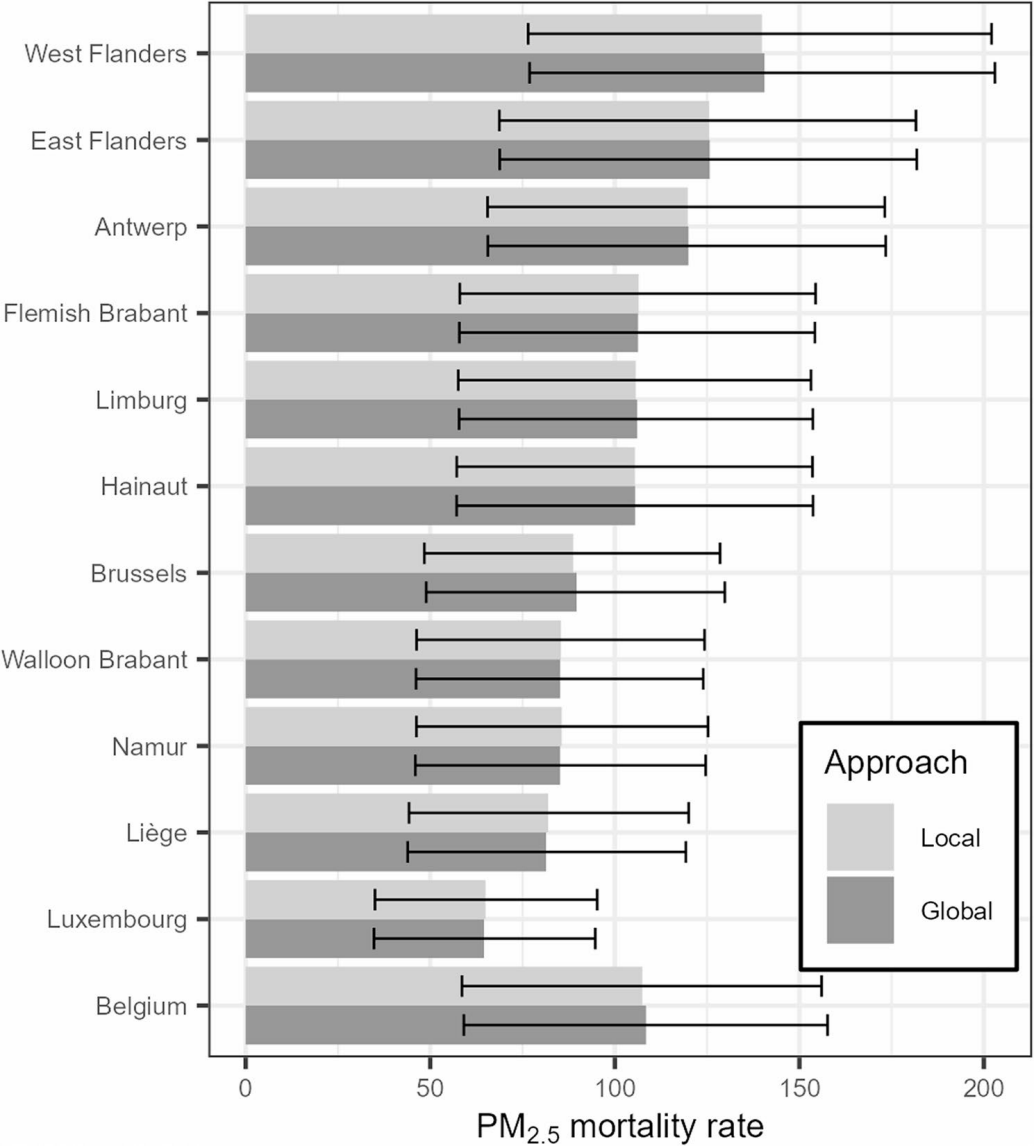
PM_2.5_-attributable all-cause mortality rate (per 100,000 inhabitants) in the Belgian provinces. For each province, the attributable mortality is calculated with both the local and global approach. The Belgian average is included for comparison

**Fig. 6:**
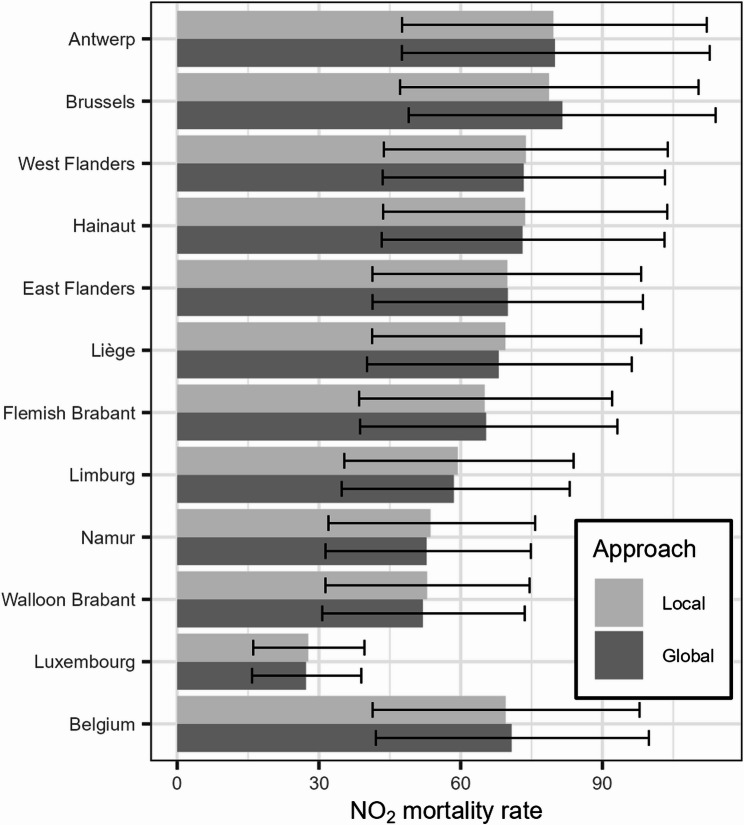
NO_2_-attributable all-cause mortality rate (per 100,000 inhabitants) in the Belgian provinces. For each province, the attributable mortality is calculated with both the local and global approach. The Belgian average is included for comparison

Aggregated to a national total, the local method estimates (95% CI) 12,276 (6,695; 17,826) deaths for PM_2.5_ and 7,944 (4,725; 11,181) for NO_2_, corresponding to a mortality rate of 107.4 (58.6; 156.0) and 69.5 (41.4; 97.9) per 100,000 persons respectively (Tables S4-S7). Figures 5 and 6 reveal important subnational differences in both PM_2.5_ and NO_2_ attributable deaths. Provinces in the northern Flemish region suffer a higher PM_2.5_ burden compared to the southern Walloon region, which could be expected given the regional imbalance in concentrations (Fig. 2). Three out of five Flemish provinces score above the Belgian average rate, while the remaining show rates close to this average. PM_2.5_-related mortality is distributed unequally within Belgium. The attributable mortality rate in the top province of West Flanders is more than double that of the bottom province Luxembourg. The Brussels-Capital Region is in the bottom half, with a PM_2.5_ mortality rate below the Belgian average.

Subnational differences are even larger for NO_2_ mortality. As could be expected from the map in Fig. 3, and confirmed by the boxplot in Fig. 4, the spatial variation in the dispersion of this pollutant is more substantial compared to PM_2.5_. Unlike the situation for PM_2.5_, a clear north-south imbalance lacks for NO_2_ mortality. The highly urbanised areas Brussels and Antwerp lead, with NO_2_ mortality rates almost triple that of Luxembourg at the bottom.

To assess any systematic bias, the aggregated local results in each province and the Belgian total are paired with estimates obtained with a global approach. The difference between the locally and globally estimated mortality rate for Belgium is about 1 death per 100,000 persons for both PM_2.5_ and NO_2_, corresponding to a deviation of approximately 1% and 2% respectively. The aggregated local results do not yield estimates that are consistently above or below the global values. The average deviation of rates in the provinces is 0.01% for PM_2.5_ and 0.48% for NO_2_. This indicates that the local approach is not biased by under- or overestimating the burden compared to the global approach. In the regions and provinces, the relative difference never surpasses 1% for PM_2.5_ and never surpasses 4% for NO_2_, where the largest difference is 3.6% in the Brussels Capital Region.

The discrepancies between the local estimates aggregated to province totals and the corresponding global estimates are limited compared to the width of the confidence intervals (Figs. 5 and 6). Comparing the confidence intervals of the local and global approach, these are similar in terms of width and position. The average difference in the width of PM_2.5_ CIs is a rate of 0.07, ranging from − 0.79 to 0.42. For NO_2_, the differences are slightly larger, ranging from − 1.78 to 0.91 in terms of rate, with an average of 0.07.

**Fig. 7:**
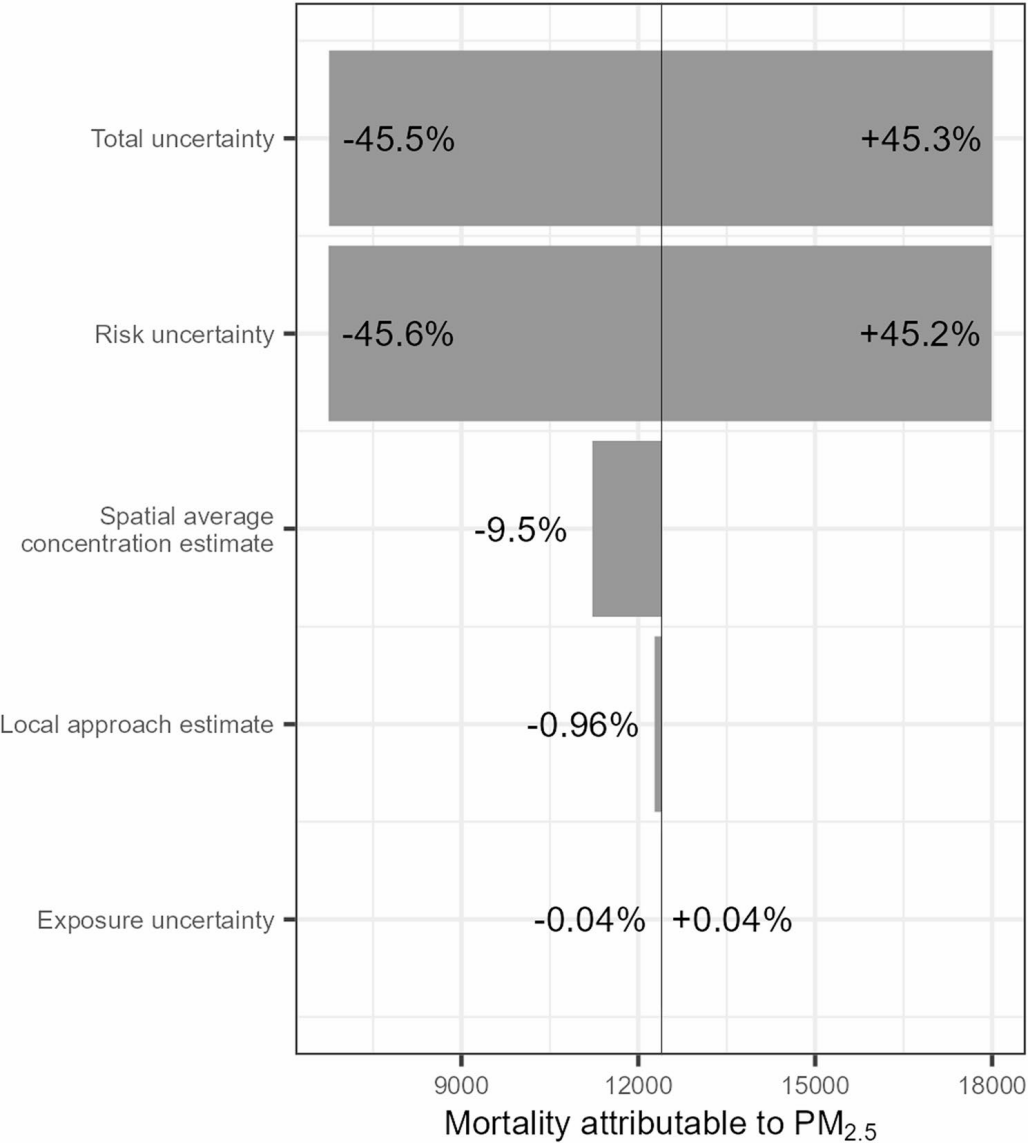
Tornado plot of all-cause mortality attributable to PM_2.5_ in Belgium. The global estimate serves as the baseline, represented by the vertical line

**Fig. 8:**
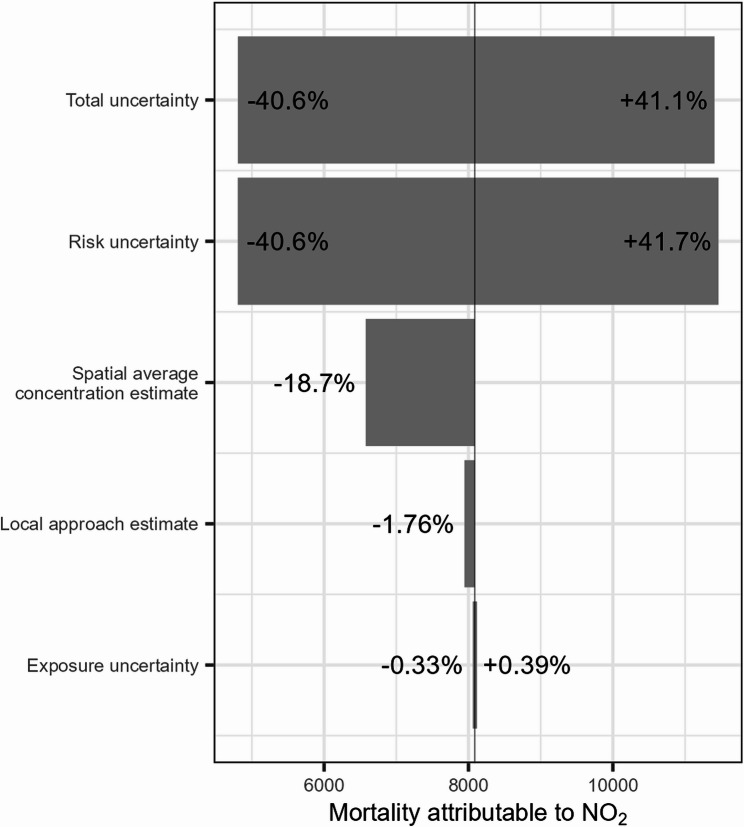
Tornado plot of all-cause mortality attributable to NO_2_ in Belgium. The global estimate serves as the baseline, represented by the vertical line

To put the magnitude of the discrepancy between the local aggregated and global estimates into perspective, it was compared with the deviation from the central estimate resulting from different sources of uncertainty and methodological choices (Tables S4-S7). The tornado plots in Figs. 7 and 8 visualise the contribution of these sources to the total uncertainty on the central global estimate for Belgium, which is taken as the baseline. The resulting spread is compared with the discrepancy between the global and local approach. To visualise the impact of population weighting in the exposure assessment, the central estimate of un-weighted spatial average concentration is included as well.

For both PM_2.5_ and NO_2_, the variability of the CRF is by far the largest contributor to the total uncertainty. Compared to the confidence interval related to risk uncertainty, the contribution of other sources appears limited. The second largest deviation relates to the use of a spatial average concentration as exposure measure, which is the case for both pollutants. In this scenario, mortality is underestimated compared to both the local approach and the global approach with population weighting. This indicates that in Belgium as a whole, the population tends to reside in areas that are on average more polluted. This is to be expected, as human activities such as road transport, residential heating, and agriculture and livestock are responsible for a large share of PM_2.5_ and NO_2_ emissions [20]. The underestimation of using a spatial average concentration is larger for NO_2_ compared to PM_2.5_, in both absolute as relative terms: 1,510 deaths or 18.7% compared to 1,175 or 9.5%. The discrepancy between the local and the global approach is larger only compared to the confidence interval on the population exposure value. Uncertainty of exposure to NO_2_ is larger, likely reflecting the greater variability of this pollutant.

## Discussion

In this study we explored a novel approach to attributable disease burden, where the estimates are derived locally at the level of statistical sectors. As a case, we estimated all-cause mortality attributable to air pollution in Belgium, as long-term exposure to PM_2.5_ and NO_2_. In contrast to the common global approach, where the burden of disease is estimated on a national or regional scale, a local approach allows to examine how the burden varies in space and between communities. We found that air pollution-attributable mortality is highly variable in Belgium, as a result of differences in local exposure and population sizes. Although PM_2.5_-attributable mortality is generally higher, the highest local exposures and attributable fractions are observed for NO_2_.

For a comparison with the global approach, the local estimates were aggregated to a national level and different subnational levels. The difference between the aggregated and global results is limited to under 2% for Belgium, and never surpasses 4% in the individual regions and provinces. The local approach does not systematically over- or underestimate the burden, which indicates that the method is not biased compared to the global approach. Both the local and global approach yield similar confidence intervals. The convergence between the global and aggregated results also ensures consistency, avoiding a confusing scenario in which the approaches would yield contradicting estimates.

The discrepancy between the approaches was compared to other methodological choices and to sources of uncertainty in a sensitivity analysis, where the global estimate for Belgium was chosen as baseline. Risk uncertainty related to the CRF was by far the greatest contributor to total uncertainty, whereas the contribution of exposure assessment was negligible. A spatial average concentration for population exposure underestimates the attributable mortality, where the underestimation is greater for NO_2_. The difference with the local estimate is limited, slightly larger compared to the population exposure uncertainty. As exposure uncertainty was very limited, the total uncertainty on the global estimate was approximately equal to the risk uncertainty. For NO_2_, risk uncertainty was slightly greater than total uncertainty. This unintuitive result is likely due to the random pairing of bootstrapped RR and exposure values, in this case leading to a narrowing of the confidence interval instead of a widening.

The sensitivity analysis did not account for all methodological choices that go into attributable burden estimation, such as the modelling approach and resolution of the air quality data, the selection of the CRF, the value of the exposure threshold, and the use of all-cause as opposed to cause-specific mortality as health outcome. The air quality data taken for exposure assessment results from the ATMO-Street model. Although validation has shown this model generally preforms well [17, 21], pollutant concentrations are not fully accurately modelled across the whole domain. This results in an additional uncertainty on the exposure assessment which has not been taken into account in the sensitivity analysis. In their European multi-city health impact assessment of PM_2.5_ and NO_2_ mortality, Khomenko et al. [22] found that their estimates were most sensitive to the choice of the CRF, and less to baseline mortality values and exposure assessment method. In their study of the burden of disease due to PM_2.5_ in Germany, Tobollik et al. [23] identified the air pollution input data, baseline mortality and the CRF selection as important factors explaining the difference between their results and estimates from other sources. In a study for China, Bai et al. [24] demonstrated how the population-weighted mean PM_2.5_ concentration decreases at coarser resolution, leading to lower attributable burden estimates.

The CRFs used in this study are based on effect estimates derived in the ELAPSE project: a RR of all-cause mortality per 10 µg m^−3^ increase in exposure concentration of 1.118 (1.060; 1.179) for PM_2.5_ and 1.045 (1.026; 1.065) for NO_2_ [18]. These relative risks are the combination of the results from a large pooled cohort study– consisting of eight individual cohorts from six EU countries– and seven separate large nationwide or citywide administrative cohorts. Exposure assessment was conducted consistently over all cohorts by relying on hybrid land use regression models using satellite observations, chemical transport models, land use data and traffic data as predictors. All effect measures are adjusted for smoking and other relevant confounding factors. The resulting combined effect estimates compile the most comprehensive evidence of air pollution health impacts in Europe up to date [19].

Aside from ELAPSE, other publications were considered as the source for CRFs. The *Health risks of air pollution in Europe* (HRAPIE) project [25] recommends the use of the all-cause mortality RRs from the meta-analysis performed by Hoek et al. [26]: a RR per 10 µg m^−3^ of 1.062 (1.040; 1.083) for PM_2.5_ and 1.055 (1.031; 1.080) for NO_2_ (for concentrations above 20 µg m^−3^). As their systematic literature review considers evidence up to January 2013, more recent studies on adverse air pollution effects published over the last ten years are not included in their summary effects. To support the development of the WHO’s 2021 Global Air Quality Guidelines, systematic reviews and meta-analyses were performed to assess the health effects of long-term exposure to particulate matter, NO_2_, ozone, sulfur dioxide and carbon monoxide [27]. An all-cause mortality RR per 10 µg m^−3^ of 1.08 (1.06; 1.09) was found for PM_2.5_ [28] and 1.02 (1.01; 1.04) for NO_2_ [29]. These reviews include studies conducted globally, and as such consider regions with exposure ranges that differ widely from those observed in Europe. Additionally, exposure assessment in some of the included studies is based on linking participants to monitoring stations, or on land use regression models with a coarse resolution compared to the exposure models used in ELAPSE. The updated WHO reviews, not yet published at the time of conducting the analyses, found higher RRs that are closer to the values of ELAPSE: 1.095 (1.064; 1.127) for PM_2.5_ [30] and 1.05 (1.03; 1.07) for NO_2_ [31]. The Global Burden of Disease Study (GBD) [32] derives CRFs for their risk factor attributable burden, but given the study’s cause-specific approach for health impact assessment, no effect estimates for all-cause mortality are available. In light of these considerations, it was decided that ELAPSE was most appropriate as source for CRFs in this study.

Several global estimates for Belgium in 2019 are available. The GBD estimates 3,659 deaths for PM_2.5_ and zero deaths for NO_2_ [33]. For both pollutants, population exposure is determined as a population-weighted annual average concentration [32]. As asthma incidence is the only outcome included for NO_2_, NO_2_-attributable mortality is zero by design. PM_2.5_-attributable mortality is considerably lower compared to this study’s estimate (12,276 deaths), in large part due to the cause-specific approach of the GBD. The European Environment Agency (EEA) reports 6,500 premature deaths for PM_2.5_ (rounded to nearest 100) and 750 for NO_2_ (rounded to nearest 10) [34], based on a population-weighted mean exposure value as explained in the report on the European air quality maps [35]. These numbers too are lower compared to this study. One reason for the discrepancy is the use of CRFs from the HRAPIE project. The RR for PM_2.5_ is lower compared to the pooled ELAPSE effect estimate. The RR for NO_2_ is slightly steeper, but the EEA instated a 20 µg m^−3^ exposure threshold. Additionally, only the mortality in adults aged 30 years and older is taken into account.

Although apparently quite rare, some examples of a similar local approach to attributable disease burden exist in the literature. In their report for Public Health England, Gowers et al. [36] calculated PM_2.5_-related mortality in the UK at the level of local authority district. In a study on the burden due to transportation noise in England, Jephcote et al. [37] provided estimates down to the local authority district level and aggregations to regional and national levels. In both studies, the authors observed a large degree of spatial variation across local districts in terms of exposure, PAFs and attributable burden. Although the scale in these studies is already quite refined compared to a national total, the local districts are large compared to the Belgian statistical sectors, with populations in the range of thousands of inhabitants. This makes the approach less suited for flexible, ad hoc aggregation. Khomenko et al. [22] estimated annual preventable deaths due to air pollution in 969 cities and 47 greater cities in Europe. They used a bottom-up approach by scaling city-level age-specific natural mortality rates according to the population in a 250 × 250 m^2^ grid, calculating an attributable fraction in each grid cell in function of the local concentration, and subsequently aggregating to the city total. Although this can be considered a local approach, it differs from the method of this study in the sense that the local mortality counts are estimated based on city-level (or if not available, regional or national) rates and a population density map, instead of the actual mortality and population in designated administrative units.

A local approach to burden of disease offers advantages over the global approach. A key strength is the flexibility to aggregate local results to obtain estimates for larger areas and populations. In contrast to national or regional studies, the local approach allows to internally compare areas and population groups. This aggregation can be implemented in a flexible ad hoc manner, not necessarily restricted to established administrative boundaries. Instead, it allows to investigate virtually any particular area of interest that can be approximated by a selection of statistical sectors. Aggregation is not limited to contiguous spatial entities, and can be according to certain characteristics of the statistical sector’s territory or the local population. For example, sectors can be aggregated based on spatial or geographical features such land use or land cover. This allows to aggregate depending on the presence of agricultural or industrial activity, adjacency of infrastructure such as highways, railways or airports, access to green space, make an urban versus rural distinction, and the like.

Statistical sectors can be aggregated based on individual-level variables in the baseline burden data, such as personal information recorded in the mortality register. As such, a local approach can be applied to different demographic groups allowing for a stratification of the estimates based on for instance age, sex or ethnicity. As such, a local approach enables to study the impacts of air pollution or other risk factors in vulnerable population groups, such as infants, children and the elderly. In case individual level data are lacking, aggregation can be based on area-level variables. In the case of the Belgian statistical sectors, socio-economic indicators are made available on this level, such as income, education and employment. Alternatively, sectors can be categorised according to an area-level index of socio-economic status or deprivation. In the case of Belgium, an index of multiple deprivation exists on the statistical sector level, based on income, education, employment, housing, health and crime [38]. Based on the combined score in these domains, the sectors are ranked and assigned to deciles, which determines the overall multiple deprivation index. Similar to the approach in Otavova et al. [39] to quantify deprivation level-specific premature mortality, we plan to examine potential social inequalities in air pollution-related burden in a follow-up article.

A local approach comes with certain limitations. There are potential issues to applying the CRF– a statistical relationship - on the scale of statistical sectors. A first issue arises when the RR of the studied outcome is subject to confounding. If the distribution of confounding factors in the local population is different from that of the general population, applying the CRF will lead to biased results [5]. A second issue relates to sectors with small populations, where there are only a few annual deaths. In this case, applying a CRF amounts to ecological fallacy (explanation see e.g [40]). For these reasons, a minimal level of aggregation might be required for the results to be meaningful. Putting a number on the minimum population needed is not straightforward, as this requires knowledge on the distribution of all relevant confounders. Aside from aggregating in space, as was the approach pursued in this study, aggregation could be accomplished in time by combining multiple years. This could be a suited approach to obtain reliable estimates for small geographic areas, but requires to sacrifice detail in the temporal dimension.

Another issue relates to the use of a log-linear CRF, which can lead to attributable fractions that are possibly unrealistically large in sectors with high local concentrations. This could be solved by using an integrated non-linear dose-response curve, where the RR increase is adapted to the level of exposure and usually levels off at high concentrations. Aggregation to large areas can also mitigate this effect, and this study did not find an overestimation of aggregated local estimate compared to the global approach.

Another limitation relates to the estimation of population exposure uncertainty in the aggregated areas. In the bootstrapping, resampled local concentrations are aggregated based on the random order of generation. This neglects any correlation in exposure between adjacent or closely spaced sectors, which might lead to an underestimation of the population-weighted average concentration in the area. The same problem arises when aggregating local burden estimates, where the uncertainty on the aggregated estimate will not fully reflect the exposure uncertainty. Dealing with this will require more sophisticated methods that take into account autocorrelation.

A final caveat relates to the difficulty in obtaining exposure and health outcome data on a local level. Data on health outcomes (e.g., total or cause-specific mortality, disease prevalence) might be relatively easy to retrieve at a national or regional level, but the availability generally decreases at increasing resolution. Obtaining such data on a sufficiently detailed level might require a linkage of several databases, involving a formal request to the data protection authority, which can be time consuming.

Policy-oriented research could benefit from a local approach to burden of disease. The bottom-up aggregation offers the advantage of easily generating estimates for specific areas or population groups of interest. This can be accomplished on an ad hoc basis, which enables to anticipate specific requests for burden estimates. The burden of disease and the impact of risk factors can be outlined for public health-related administrative areas, such as those related to the organisation of health care or the provision of social benefits. Such assessment amounts to the monitoring of the health of the population on a more refined scale. This could help local authorities in setting priorities, and be used to inform the public about the state of air quality and related health issues in their community. Prevention or intervention measures can be tailored to a particular local situation, and scarce resources could be allocated more efficiently to where it would be most beneficial.

## Conclusions

We explored a local approach to estimate all-cause mortality attributable to air pollution in Belgium. A local assessment allows to map the burden of disease and explore differences between geographic areas, which this study showed can be substantial. Relying on general population CRFs raised concerns for estimating the attributable mortality in small distinct populations. However, aggregating local estimates to different subnational levels indicated no systematic bias compared to the usual global approach. The discrepancy between aggregated and global estimates is also limited compared to the effect of other methodological choices and different sources of uncertainty, as was shown in a sensitivity analysis. A local approach is suited to obtain estimates in a bottom-up fashion depending on specific needs of policymakers and other stakeholders. Such assessment offers additional possibilities, for example to examine whether socioeconomic inequalities exist in the distribution of air quality burden.

## Supplementary Information

Supplementary Material 1.

## Data Availability

The data that support the findings of this study are available from Statbel but restrictions apply to the availability of these data, which were used under license for the current study, and so are not publicly available. Data requests to Statbel can be initiated by contacting their office at statbel@economie.fgov.be. More information on requesting individual-level data from Statbel can be found here: https://statbel.fgov.be/en/about-statbel/what-we-do/microdata-research.

## Abbreviations

CRA: comparative risk assessment
CRF: concentration-response function
EEA: European Environment Agency
GBD: Global Burden of Disease Study
IFDM: immission frequency distribution model
NO_2_: nitrogen dioxide
OSPM: operational street pollution model
PAF: population attributable fraction
PM_2.5_: particulate matter with a diameter ≤ 2.5 μm
RIO: residual interpolation optimised for ozone
RR: relative risk

## References

[1] Devleesschauwer B, Maertens de Noordhout C, Smit GSA, Duchateau L, Dorny P, Stein C, et al. Quantifying burden of disease to support public health policy in belgium: opportunities and constraints. BMC Public Health. 2014;14(1):1196. 25416547 10.1186/1471-2458-14-1196 PMC4246467

[2] Murray CJ, Ezzati M, Lopez AD, Rodgers A, Vander Hoorn S. Comparative quantification of health risks: conceptual framework and methodological issues. Popul Health Metrics. 2003;1(1):1. 10.1186/1478-7954-1-1 PMC156894 12780936

[3] Barendregt JJ, Veerman JL. Categorical versus continuous risk factors and the calculation of potential impact fractions. J Epidemiol Community Health. 2010;64(3):209–12. 19692711 10.1136/jech.2009.090274

[4] Plass D, Hilderink H, Lehtomäki H, Øverland S, Eikemo TA, Lai T, et al. Estimating risk factor attributable burden – challenges and potential solutions when using the comparative risk assessment methodology. Arch Public Health. 2022;80(1):148. 35624479 10.1186/s13690-022-00900-8 PMC9137119

[5] Steenland K, Armstrong B. An overview of methods for calculating the burden of disease due to specific. Risk Factors: Epidemiol. 2006;17(5):512–9. 10.1097/01.ede.0000229155.05644.43 16804473

[6] Ferguson J, Alvarez-Iglesias A, Newell J, Hinde J, O’Donnell M. Estimating average attributable fractions with confidence intervals for cohort and case–control studies. Stat Methods Med Res. 2018;27(4):1141–52. 27342575 10.1177/0962280216655374

[7] Greenland S. Interval Estimation by simulation as an alternative to and extension of confidence intervals. Int J Epidemiol. 2004;33(6):1389–97. 15319402 10.1093/ije/dyh276

[8] Counil E. Contribution of causal factors to disease burden: how to interpret attributable fractions. Breathe. 2021;17:210086. 10.1183/20734735.0086-2021 PMC8753648 35035565

[9] Rowe AK, Powell KE, Flanders WD. Why population attributable fractions can sum to more than one. Am J Prev Med. 2004;26(3):243–9. 15026106 10.1016/j.amepre.2003.12.007

[10] Eurostat. Population density by NUTS 3 region [Internet]. 2024 [cited 2024 Sep 18]. Available from: 10.2908/DEMO_R_D3DENS

[11] Fierens F, Vanpoucke C, Trimpeneers E, Dumoulin R, Maetz P, Hutsemékers V, et al. Jaarrapport Luchtkwaliteit in België 2022. IRCEL - CELINE; 2023. https://www.irceline.be/nl/documentatie/publicaties/jaarrapporten/jaarrapport-luchtkwaliteit-in-belgie-2022/at_download/file.

[12] Demoury C, Aerts R, Berete F, Lefebvre W, Pauwels A, Vanpoucke C, et al. Impact of short-term exposure to air pollution on natural mortality and vulnerable populations: a multi-city case-crossover analysis in Belgium. Environ Health. 2024;23(1):11. 38267996 10.1186/s12940-024-01050-w PMC10809644

[13] Vranken A, Bijnens E, Horemans C, Leclercq A, Kestens W, Karakaya G, et al. Association of air pollution and green space with all-cause general practitioner and emergency room visits: A cross-sectional study of young people and adults living in Belgium. Environ Res. 2023;236:116713. 37481061 10.1016/j.envres.2023.116713

[14] Pelgrims I, Devleesschauwer B, Guyot M, Keune H, Nawrot TS, Remmen R, et al. Association between urban environment and mental health in brussels, Belgium. BMC Public Health. 2021;21(1):635. 33794817 10.1186/s12889-021-10557-7 PMC8015067

[15] Statbel. Statistische sectoren [Internet]. Statbel. Available from: https://statbel.fgov.be/nl/over-statbel/methodologie/classificaties/statistische-sectoren

[16] IRCEL-CELINE. Air quality models [Internet]. Belgian Interregional Environment Agency (IRCEL - CELINE). [cited 2024 Sep 3]. Available from: https://www.irceline.be/en/documentation/models

[17] Lefebvre W, Van Poppel M, Maiheu B, Janssen S, Dons E. Evaluation of the RIO-IFDM-street Canyon model chain. Atmos Environ. 2013;77:325–37.

[18] Hoffmann B, Brunekreef B, Andersen ZJ, Forastiere F, Boogaard H. Benefits of future clean air policies in Europe: Proposed analyses of the mortality impacts of PM: 2.5: and NO: 2. Environmental Epidemiology [Internet]. 2022;6(5). Available from: https://journals.lww.com/environepidem/fulltext/2022/10000/benefits_of_future_clean_air_policies_in_europe_.5.aspx 10.1097/EE9.0000000000000221 PMC9556041 36249272

[19] Brunekreef B, Strak M, Chen J, Andersen ZJ, Atkinson R, Carey I et al. Mortality and morbidity effects of Long-Term exposure to Low-Level PM2.5, BC, NO2, and O3: an analysis of European cohorts in the ELAPSE Project. 2021;150. PMC9476567 36106702

[20] European Environment Agency. National air pollutant emissions data viewer 2005–2022 [Internet]. 2024 [cited 2024 Sep 3]. Available from: https://www.eea.europa.eu/en/topics/in-depth/air-pollution/national-air-pollutant-emissions-data-viewer-2005-2022?activeTab=570bee2d-1316-48cf-adde-4b640f92119b

[21] Hooyberghs H, De Craemer S, Lefebvre W, Vranckx S, Maiheu B, Trimpeneers E, et al. Validation and optimization of the ATMO-Street air quality model chain by means of a large-scale citizen-science dataset. Atmos Environ. 2022;272:118946.

[22] Khomenko S, Cirach M, Pereira-Barboza E, Mueller N, Barrera-Gómez J, Rojas-Rueda D, et al. Premature mortality due to air pollution in European cities: a health impact assessment. Lancet Planet Health. 2021;5(3):e121–34. 33482109 10.1016/S2542-5196(20)30272-2

[23] Tobollik M, Kienzler S, Schuster C, Wintermeyer D, Plass D. Burden of disease due to ambient particulate matter in Germany—Explaining the differences in the available estimates. IJERPH. 2022;19(20):13197. 36293778 10.3390/ijerph192013197 PMC9602590

[24] Bai H, Wu H, Gao W, Wang S, Cao Y. Influence of Spatial resolution of PM2.5 concentrations and population on health impact assessment from 2010 to 2020 in China. Environ Pollut. 2023;326:121505. 36965685 10.1016/j.envpol.2023.121505

[25] WHO Regional Office for Europe. Health risks of air pollution in Europe – HRAPIE project: Recommendations for concentration–response functions for cost–benefit analysis of particulate matter, ozone and nitrogen dioxide. Copenhagen. 2013. p. 60. WHO/EURO:2013-6696-46462-67326.

[26] Hoek G, Krishnan RM, Beelen R, Peters A, Ostro B, Brunekreef B, et al. Long-term air pollution exposure and cardio- respiratory mortality: a review. Environ Health. 2013;12(1):43. 23714370 10.1186/1476-069X-12-43 PMC3679821

[27] World Health Organization. WHO global air quality guidelines: particulate matter (PM2.5 and PM10), ozone, nitrogen dioxide, sulfur dioxide and carbon monoxide [Internet]. Geneva: World Health Organization. 2021 [cited 2022 Jul 5]. Available from: https://apps.who.int/iris/handle/10665/345329 34662007

[28] Chen J, Hoek G. Long-term exposure to PM and all-cause and cause-specific mortality: A systematic review and meta-analysis. Environ Int. 2020;143:105974. 32703584 10.1016/j.envint.2020.105974

[29] Huangfu P, Atkinson R. Long-term exposure to NO2 and O3 and all-cause and respiratory mortality: A systematic review and meta-analysis. Environ Int. 2020;144:105998. 33032072 10.1016/j.envint.2020.105998 PMC7549128

[30] Orellano P, Kasdagli MI, Pérez Velasco R, Samoli E. Long-Term exposure to particulate matter and mortality: an update of the WHO global air quality guidelines systematic review and Meta-Analysis. Int J Public Health. 2024;69:1607683. 39399882 10.3389/ijph.2024.1607683 PMC11466858

[31] Kasdagli MI, Orellano P, Pérez Velasco R, Samoli E. Long-Term exposure to nitrogen dioxide and Ozone and mortality: update of the WHO air quality guidelines systematic review and Meta-Analysis. Int J Public Health. 2024;69:1607676. 39494092 10.3389/ijph.2024.1607676 PMC11527649

[32] Brauer M, Roth GA, Aravkin AY, Zheng P, Abate KH, Abate YH, et al. Global burden and strength of evidence for 88 risk factors in 204 countries and 811 subnational locations, 1990–2021: a systematic analysis for the global burden of disease study 2021. Lancet. 2024;403(10440):2162–203. 38762324 10.1016/S0140-6736(24)00933-4 PMC11120204

[33] Global Burden of Disease Collaborative Network. Global Burden of Disease Study 2021. (GBD 2021) Results [Internet]. Seattle, United States: Institute for Health Metrics and Evaluation (IHME); 2022. Available from: https://vizhub.healthdata.org/gbd-results/

[34] González Ortiz A, Gsella A, Guerreiro, Cristina, Soares J, Horálek J. Health risk assessments of air pollution: Estimations of the 2019 HRA, benefit analysis of reaching specific air quality standards and more. European Topic Centre on Air pollution, transport, noise and industrial pollution; 2021 Nov. Report No.: ETC/ATNI 2021/10. https://www.eionet.europa.eu/etcs/etc-atni/products/etc-atni-reports/etc-atni-report-10-2021-health-risk-assessments-of-air-pollution-estimations-of-the-2019-hra-benefit-analysis-of-reaching-specific-air-quality-standards-and-more.

[35] Horálek J, Vlasáková L, Schreiberová M, Marková J, Schneider P, Kurfürst P, et al. European air quality maps for 2019. PM10, PM2.5, Ozone, NO2 and NOx. Spatial estimates and their uncertainties. European Topic Centre on Air pollution, transport, noise and industrial pollution; 2021 Sep. Report No.: ETC/ATNI 2021/1. https://www.eionet.europa.eu/etcs/etc-atni/products/etc-atni-reports/etc-atni-report-1-2021-european-air-quality-maps-for-2019-pm10-pm2-5-ozone-no2-and-nox-spatial-estimates-and-their-uncertainties.

[36] Gowers AM, Miller BG, Stedman JR. Estimating local mortality burdens associated with particulate air pollution. Didcot, Oxfordshire: Centre for Radiation, Chemical and Environmental Hazards, Public Health England;: Chilton; 2014.

[37] Jephcote C, Clark SN, Hansell AL, Jones N, Chen Y, Blackmore C, et al. Spatial assessment of the attributable burden of disease due to transportation noise in England. Environ Int. 2023;178:107966. 10.1016/j.envint.2023.107966 37390771

[38] Otavova M, Faes C, Bouland C, De Clercq E, Vandeninden B, Eggerickx T, et al. Inequalities in mortality associated with housing conditions in Belgium between 1991 and 2020. BMC Public Health. 2022;22(1):2397. 36539802 10.1186/s12889-022-14819-w PMC9769013

[39] Otavova M, Masquelier B, Faes C, van den Borre L, Vandeninden B, de Clercq E, et al. Trends in socioeconomic inequalities in cause-specific premature mortality in belgium, 1998–2019. BMC Public Health. 2024;24(1):470. 38355531 10.1186/s12889-024-17933-z PMC10868013

[40] Wakefield J. Ecologic studies revisited. Annu Rev Public Health. 2008;29(1):75–90. 17914933 10.1146/annurev.publhealth.29.020907.090821

