# Supplementary material for: Local approach to attributable disease burden: a case study on air pollution and mortality in Belgium
Source: BMC Public Health. 2025 Jul 12;25:2439. doi: 10.1186/s12889-025-23625-z (PMC12255123; doi:10.1186/s12889-025-23625-z)
Supplement: Supplementary file 1 — Supplementary Material 1. [file 12889_2025_23625_MOESM1_ESM.docx]

Local approach to attributable disease burden: a case study on air pollution and mortality in Belgium
-
Supplementary materials

Arno Pauwels^1,2*^, Claire Demoury^1^, Eva M. De Clercq^1^, Brecht Devleesschauwer^2,3^

*^a^ Department of Chemical and Physical Health Risks, Sciensano, Victor Hortaplein 40, 1060 Sint-Gillis, Belgium*

*^b^ Department of Epidemiology and Public Health, Sciensano, Rue Ernest Blerot 1, 1070 Anderlecht, Belgium*

*^c^ Department of Translational Physiology, Infectiology and Public Health, Ghent University, Salisburylaan 133, 9820 Merelbeke, Belgium*

* Corresponding author

Arno Pauwels, MSc

Health information, Department of Epidemiology and Public Health, Sciensano, Rue Ernest Blerot 1, 1070 Anderlecht, Belgium.

Tel.: + 32 2 642 51 15 | E-mail: [arno.pauwels@sciensano.be](mailto:arno.pauwels@sciensano.be)

**Email addresses**

AP: [arno.pauwels@sciensano.be](mailto:arno.pauwels@sciensano.be)

CD: [claire.demoury@sciensano.be](mailto:claire.demoury@sciensano.be)

EDC: [eva.declercq@sciensano.be](mailto:eva.declercq@sciensano.be)

BD: [brecht.devleesschauwer@sciensano.be](mailto:brecht.devleesschauwer@sciensano.be)

Table S1: Summary statistics of local baseline total mortality data and of local attributable mortality estimates for PM_2.5_ and NO_2_ in the statistical sectors (N = 19,414).

|  | Mean | SD | IQR | Min | Q1 | Median | Q3 | Max |
| --- | --- | --- | --- | --- | --- | --- | --- | --- |
| Population | 588.5 | 719.3 | 664 | 1 | 120 | 336 | 784 | 8340 |
| Mortality | 5.6 | 9.3 | 5 | 0 | 1 | 2 | 6 | 139 |

SD: standard deviation; IQR: interquartile range; Q1: first quartile; Q3: third quartile.

Table S2: Summary statistics of local baseline total mortality data and of local attributable mortality estimates for PM_2.5_ in the statistical sectors (N = 19,414).

|  | Mean | SD | IQR | Min | Q1 | Median | Q3 | Max |
| --- | --- | --- | --- | --- | --- | --- | --- | --- |
| Exposure | 9.7 | 2.6 | 4.8 | 4.8 | 7.3 | 9.9 | 12.1 | 14.7 |
| PAF | 10.3 | 2.6 | 4.8 | 5.3 | 7.9 | 10.5 | 12.7 | 15.2 |
| Attributable mortality | 0.6 | 1.1 | 0.6 | 0.0 | 0.1 | 0.2 | 0.7 | 19.2 |

SD: standard deviation; IQR: interquartile range; Q1: first quartile; Q3: third quartile.

Table S3: Summary statistics of local baseline total mortality data and of local attributable mortality estimates for NO_2_ in the statistical sectors (N = 19,414).

|  | Mean | SD | IQR | Min | Q1 | Median | Q3 | Max |
| --- | --- | --- | --- | --- | --- | --- | --- | --- |
| Exposure | 14.0 | 5.6 | 5.9 | 3.9 | 10.8 | 13.9 | 16.7 | 43.8 |
| PAF | 6.0 | 2.3 | 2.5 | 1.7 | 4.7 | 6.0 | 7.1 | 17.5 |
| Attributable mortality | 0.4 | 0.8 | 0.4 | 0.0 | 0.02 | 0.1 | 0.4 | 14.5 |

*SD: standard deviation; IQR: interquartile range; Q1: first quartile; Q3: third quartile.*

Table S4: Global and aggregated estimates (95% CI) of all-cause deaths attributable to PM_2.5_ (rounded to the nearest integer). Table compares the global approach (baseline) to the local approach and the three other scenarios included in the sensitivity analysis.

|  | Local approach | Global approach | Spatial average concentration | Risk uncertainty | Exposure uncertainty |
| --- | --- | --- | --- | --- | --- |
| Antwerp | 2,225 (1,217; 3,217) | 2,229 (1,219; 3,222) | 2,222 (1,215; 3,211) | 2,229 (1,220; 3,221) | 2,244 (2,241; 2,247) |
| Walloon Brabant | 345 (187; 502) | 344 (186; 500) | 340 (184; 495) | 344 (186; 501) | 346 (345; 346) |
| Hainaut | 1,417 (769; 2,064) | 1,418 (768; 2,066) | 1,363 (737; 1,987) | 1,418 (768; 2,066) | 1,426 (1,425; 1,428) |
| Liège | 907 (490; 1,329) | 900 (486; 1,320) | 842 (454; 1,234) | 901 (486; 1,319) | 906 (904; 908) |
| Limburg | 923 (503; 1,338) | 927 (505; 1,343) | 905 (493; 1,312) | 927 (505; 1,343) | 933 (932; 934) |
| Luxembourg | 185 (100; 271) | 184 (99; 269) | 175 (94; 257) | 184 (99; 269) | 185 (184; 185) |
| Namur | 423 (229; 619) | 421 (227; 616) | 394 (212; 576) | 421 (227; 615) | 423 (423; 424) |
| East Flanders | 1,902 (1,041; 2,750) | 1,904 (1,042; 2,754) | 1,867 (1,021; 2,700) | 1,904 (1,042; 2,752) | 1,917 (1,915; 1,919) |
| Flemish Brabant | 1,220 (665; 1,769) | 1,218 (664; 1,767) | 1,208 (658; 1,752) | 1,218 (664; 1,766) | 1,226 (1,224; 1,227) |
| West Flanders | 1,672 (915; 2,416) | 1,680 (919; 2,426) | 1,667 (912; 2,409) | 1,680 (920; 2,427) | 1,692 (1,690; 1,693) |
| Brussels | 1,069 (583; 1,549) | 1,080 (590; 1,565) | 1,066 (581; 1,544) | 1,080 (590; 1,565) | 1,087 (1,087; 1,087) |
| Flanders | 7,942 (4,341; 11,488) | 7,944 (4,341; 11,493) | 7,826 (4,276; 11,325) | 7,944 (4,343; 11,492) | 7,998 (7,993; 8,002) |
| Wallonia | 3,274 (1,772; 4,786) | 3,261 (1,763; 4,769) | 3,025 (1,633; 4,429) | 3,264 (1,764; 4,766) | 3,283 (3,280; 3,285) |
| Belgium | 12,276 (6,695; 17,826) | 12,395 (6,753; 18,012) | 11,219 (6,095; 16,341) | 12,408 (6,757; 18,005) | 12,487 (12,482; 12,492) |

Table S5: Global and aggregated estimates (95% CI) of PM_2.5_- attributable all-cause mortality rate (per 100,000 inhabitants, rounded to one decimal). Table compares the global approach (baseline) to the local approach and the three other scenarios included in the sensitivity analysis.

|  | Local approach | Global approach | Spatial average concentration | Risk uncertainty | Exposure uncertainty |
| --- | --- | --- | --- | --- | --- |
| Antwerp | 119.8 (65.5; 173.2) | 120.0 (65.6; 173.4) | 119.6 (65.4; 172.9) | 120.0 (65.7; 173.4) | 120.8 (120.7; 121.0) |
| Walloon Brabant | 85.4 (46.3; 124.3) | 85.2 (46.1; 124.0) | 84.2 (45.6; 122.6) | 85.2 (46.2; 124.0) | 85.7 (85.6; 85.8) |
| Hainaut | 105.5 (57.2; 153.6) | 105.5 (57.2; 153.7) | 101.4 (54.9; 147.8) | 105.5 (57.2; 153.8) | 106.1 (106.0; 106.2) |
| Liège | 81.9 (44.2; 120.0) | 81.3 (43.9; 119.3) | 76.1 (41.0; 111.5) | 81.4 (43.9; 119.1) | 81.9 (81.7; 82.0) |
| Limburg | 105.7 (57.6; 153.1) | 106.1 (57.8; 153.7) | 103.6 (56.4; 150.2) | 106.1 (57.8; 153.7) | 106.8 (106.6; 106.9) |
| Luxembourg | 65.0 (35.0; 95.2) | 64.6 (34.7; 94.7) | 61.5 (33.0; 90.2) | 64.6 (34.7; 94.7) | 64.9 (64.8; 65.0) |
| Namur | 85.6 (46.2; 125.3) | 85.1 (45.9; 124.6) | 79.6 (42.9; 116.6) | 85.2 (46.0; 124.5) | 85.6 (85.5; 85.8) |
| East Flanders | 125.6 (68.7; 181.6) | 125.7 (68.8; 181.8) | 123.3 (67.4; 178.3) | 125.7 (68.8; 181.8) | 126.6 (126.5; 126.7) |
| Flemish Brabant | 106.5 (58.0; 154.4) | 106.3 (57.9; 154.2) | 105.4 (57.5; 152.9) | 106.3 (57.9; 154.1) | 107.0 (106.9; 107.1) |
| West Flanders | 139.9 (76.5; 202.1) | 140.6 (76.9; 203.0) | 139.5 (76.3; 201.5) | 140.6 (77.0; 203.1) | 141.5 (141.4; 141.6) |
| Brussels | 88.7 (48.4; 128.5) | 89.6 (48.9; 129.8) | 88.4 (48.2; 128.1) | 89.6 (48.9; 129.8) | 90.2 (90.2; 90.2) |
| Flanders | 120.6 (65.9; 174.4) | 120.6 (65.9; 174.5) | 118.8 (64.9; 171.9) | 120.6 (65.9; 174.5) | 121.4 (121.4; 121.5) |
| Wallonia | 90.1 (48.8; 131.7) | 89.8 (48.5; 131.3) | 83.3 (45.0; 121.9) | 89.9 (48.6; 131.2) | 90.4 (90.3; 90.4) |
| Belgium | 107.4 (58.6; 156.0) | 108.5 (59.1; 157.6) | 98.2 (53.3; 143.0) | 108.6 (59.1; 157.6) | 109.3 (109.3; 109.3) |

Table S6: Global and aggregated estimates (95% CI) of all-cause deaths attributable to NO_2_ (rounded to the nearest integer). Table compares the global approach (baseline) to the local approach and the three other scenarios included in the sensitivity analysis.

|  | Local approach | Global approach | Spatial average concentration | Risk uncertainty | Exposure uncertainty |
| --- | --- | --- | --- | --- | --- |
| Antwerp | 1,479 (884; 2,082) | 1,485 (883; 2,093) | 1,352 (804; 1,908) | 1,485 (885; 2,099) | 1,489 (1,477; 1,503) |
| Walloon Brabant | 213 (127; 301) | 210 (124; 297) | 197 (117; 279) | 210 (124; 298) | 210 (208; 213) |
| Hainaut | 990 (586; 1,394) | 982 (582; 1,386) | 874 (517; 1,241) | 982 (583; 1,394) | 985 (979; 992) |
| Liège | 769 (456; 1,087) | 753 (445; 1,065) | 604 (356; 858) | 754 (447; 1,070) | 755 (747; 765) |
| Limburg | 519 (309; 733) | 512 (304; 726) | 486 (288; 689) | 512 (303; 726) | 513 (508; 518) |
| Luxembourg | 79 (46; 113) | 78 (45; 111) | 66 (39; 94) | 78 (46; 111) | 78 (77; 79) |
| Namur | 265 (158; 374) | 261 (155; 370) | 211 (125; 300) | 261 (154; 371) | 261 (258; 266) |
| East Flanders | 1,058 (626; 1,487) | 1,060 (626; 1,493) | 988 (585; 1,400) | 1,060 (629; 1,502) | 1,063 (1,054; 1,072) |
| Flemish Brabant | 746 (441; 1,055) | 749 (444; 1,068) | 722 (429; 1,024) | 749 (445; 1,062) | 751 (744; 759) |
| West Flanders | 882 (523; 1,241) | 877 (520; 1,234) | 827 (491; 1,169) | 877 (520; 1,244) | 879 (873; 885) |
| Brussels | 949 (569; 1,330) | 983 (591; 1,374) | 953 (567; 1,337) | 983 (589; 1,382) | 986 (974; 999) |
| Flanders | 4,684 (2,788; 6,605) | 4,694 (2,792; 6,634) | 4,330 (2,572; 6,133) | 4,694 (2,790; 6,651) | 4,707 (4,689; 4,728) |
| Wallonia | 2,315 (1,373; 3,270) | 2,271 (1,343; 3,214) | 1,819 (1,073; 2,578) | 2,272 (1,346; 3,229) | 2,278 (2,266; 2,291) |
| Belgium | 7,944 (4,725; 11,181) | 8,087 (4,805; 11,407) | 6,577 (3,889; 9,302) | 8,092 (4,809; 11,468) | 8,114 (8,087; 8,145) |

Table S7: Global and aggregated estimates (95% CI) of NO_2_- attributable all-cause mortality rate (per 100,000 inhabitants, rounded to one decimal). Table compares the global approach (baseline) to the local approach and the three other scenarios included in the sensitivity analysis.

|  | Local approach | Global approach | Spatial average concentration | Risk uncertainty | Exposure uncertainty |
| --- | --- | --- | --- | --- | --- |
| Antwerp | 79.6 (47.6; 112.1) | 79.9 (47.5; 112.7) | 72.8 (43.3; 102.7) | 79.9 (47.7; 113.0) | 80.2 (79.5; 80.9) |
| Walloon Brabant | 52.9 (31.4; 74.6) | 52.0 (30.7; 73.6) | 48.9 (28.9; 69.2) | 52.0 (30.8; 73.9) | 52.1 (51.6; 52.8) |
| Hainaut | 73.7 (43.6; 103.7) | 73.1 (43.3; 103.1) | 65.1 (38.5; 92.3) | 73.1 (43.4; 103.8) | 73.3 (72.8; 73.8) |
| Liège | 69.5 (41.2; 98.2) | 68.0 (40.2; 96.2) | 54.6 (32.2; 77.5) | 68.1 (40.4; 96.7) | 68.3 (67.5; 69.1) |
| Limburg | 59.4 (35.4; 83.9) | 58.6 (34.8; 83.1) | 55.6 (33.0; 78.8) | 58.6 (34.7; 83.1) | 58.7 (58.1; 59.3) |
| Luxembourg | 27.7 (16.1; 39.6) | 27.3 (15.8; 39.0) | 23.1 (13.5; 33.0) | 27.3 (16.0; 39.0) | 27.3 (26.9; 27.8) |
| Namur | 53.6 (32.0; 75.8) | 52.8 (31.4; 74.9) | 42.7 (25.3; 60.6) | 52.8 (31.2; 75.2) | 52.9 (52.3; 53.7) |
| East Flanders | 69.9 (41.3; 98.2) | 70.0 (41.3; 98.6) | 65.3 (38.6; 92.5) | 70.0 (41.6; 99.2) | 70.2 (69.6; 70.8) |
| Flemish Brabant | 65.1 (38.5; 92.1) | 65.4 (38.7; 93.2) | 63.0 (37.4; 89.4) | 65.4 (38.8; 92.7) | 65.6 (64.9; 66.3) |
| West Flanders | 73.8 (43.7; 103.8) | 73.3 (43.5; 103.2) | 69.2 (41.0; 97.8) | 73.3 (43.5; 104.0) | 73.5 (73.1; 74.1) |
| Brussels | 78.7 (47.2; 110.3) | 81.5 (49.0; 113.9) | 79.1 (47.0; 110.9) | 81.5 (48.9; 114.7) | 81.8 (80.8; 82.9) |
| Flanders | 71.1 (42.3; 100.3) | 71.3 (42.4; 100.7) | 65.7 (39.1; 93.1) | 71.3 (42.4; 101.0) | 71.5 (71.2; 71.8) |
| Wallonia | 63.7 (37.8; 90.0) | 62.5 (37.0; 88.5) | 50.1 (29.5; 71.0) | 62.5 (37.0; 88.9) | 62.7 (62.4; 63.1) |
| Belgium | 69.5 (41.4; 97.9) | 70.8 (42.1; 99.8) | 57.6 (34.0; 81.4) | 70.8 (42.1; 100.4) | 71.0 (70.8; 71.3) |
